# Reconstructive Technique in the Treatment of Merkel Cell Carcinoma of the Upper Eyelid: A Case Report

**DOI:** 10.1155/crop/8152342

**Published:** 2026-01-28

**Authors:** Ryo Yamochi, Toshiaki Numajiri

**Affiliations:** ^1^ Department of Plastic and Reconstructive Surgery, Omihachiman Community Medical Center, Omihachiman, Shiga, Japan; ^2^ Department of Plastic and Reconstructive Surgery, Kyoto Prefectural University of Medicine, Kyoto, Japan, kpu-m.ac.jp

**Keywords:** Lateral cantholysis, Lateral canthotomy, Merkel cell carcinoma, Switch flap, Upper eyelid reconstruction

## Abstract

**Background and Aims:**

Merkel cell carcinoma is a rare highly malignant disease that requires a wide resection and careful reconstruction of the resulting defect. We removed a Merkel cell carcinoma that had developed on the upper eyelid of an 86‐year‐old man and reconstructed the eyelid using a switch flap.

**Methods:**

The large size of the defect made it difficult to close the lower eyelid switch harvest site. Because of the patient’s high risk of bleeding, closure of the switch flap donor site using a malar flap and cartilage graft was not performed. Instead, lateral canthotomy and lateral cantholysis were performed, and the ear‐side end of the switch flap donor site was advanced 5 mm toward the nasal side, which allowed closure of the lower eyelid flap donor site.

**Results:**

The switch flap was detached 16 days after the initial surgery. In the 6 months since the surgery, there have been no problems with the function of the eyelids or the cosmetic appearance, or recurrence of the tumor. The results of this case suggest that this method is a good option for large full‐thickness upper eyelid defects of 13–20 mm. Lateral canthotomy with lateral cantholysis is a well‐known procedure, but there have been no reports of its combination with a switch flap.

**Conclusion:**

We believe that this method is positioned between the direct closure and malar flap, and that this method allows for a quick and minimally invasive reconstruction. The treatment of Merkel cell carcinoma requires extensive excision, and it is easy to meet the aforementioned criteria when it occurs in the upper eyelid. Because this carcinoma occurs frequently in older people, who can have a high bleeding risk, this minimally invasive method is useful for treating Merkel cell carcinoma of the upper eyelid.

## 1. Introduction

Merkel cell carcinoma is a rare disease that commonly affects the face of elderly people [1]. This carcinoma is also highly malignant and usually requires a wide resection, which makes reconstruction of the resulting defect important [2]. In the case presented here, we performed reconstruction using a switch flap after the excision of a Merkel cell carcinoma of the upper eyelid. Because the patient had a high risk of bleeding, minimally invasive surgery was desirable. We report here on eyelid reconstruction, which involved some small innovations.

## 2. Case Presentation

An 86‐year‐old man noticed a small nodule on his upper left eyelid that increased in size gradually over the next month, and he visited his previous doctor at an ophthalmology clinic. After the application of steroid and antibacterial eye ointments for 7 days without improvement, he was referred to our department for a more detailed examination. His past medical history included thrombocytopenia, angina pectoris, and chronic renal failure.

**Figure 1:**
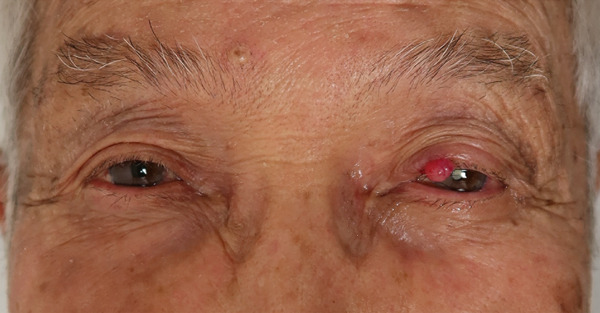
The initial examination findings. A well‐defined red dome‐shaped mass measuring 4 mm in diameter was seen on the left upper eyelid margin.

On initial examination, a well‐defined red dome‐shaped mass measuring 4 mm in diameter was seen on the left upper eyelid margin. There was no exposure of the tumor to the conjunctiva (Figure 1).

A partial biopsy was performed at the initial consultation, and the pathology findings were as follows. Hematoxylin and eosin staining showed a high nuclear–cytoplasmic ratio and strongly stained nucleated, proliferating, round cells in individual or small clusters. Immunostaining showed cells positive for cytokeratin 20 (dot‐like or comma‐like appearance in the cytoplasm), synaptophysin, and neuron‐specific enolase. Based on these results, the patient was diagnosed with upper eyelid Merkel cell carcinoma (Figure 2).

Figure 2Pathology findings: (a), Hematoxylin and eosin staining showed a high nuclear–cytoplasmic ratio and strongly stained nucleated, proliferating, round cells in individual or small clusters. (b), Cytokeratin 20 staining appeared as dot‐like or comma‐like staining in the cytoplasm. (c), Synaptophysin staining was weak but positive. (d), Neuron‐specific enolase staining was positive.

**Figure:**
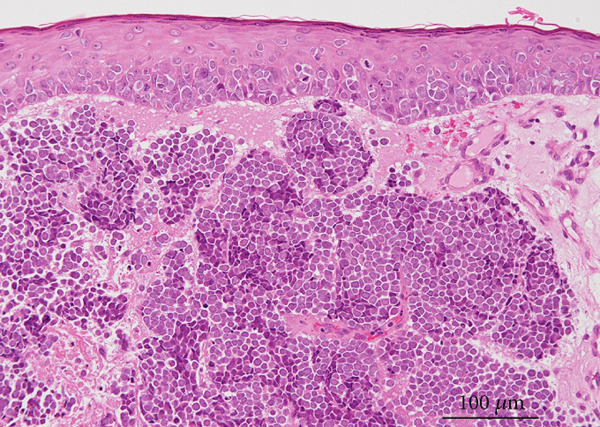
(a)

**Figure:**
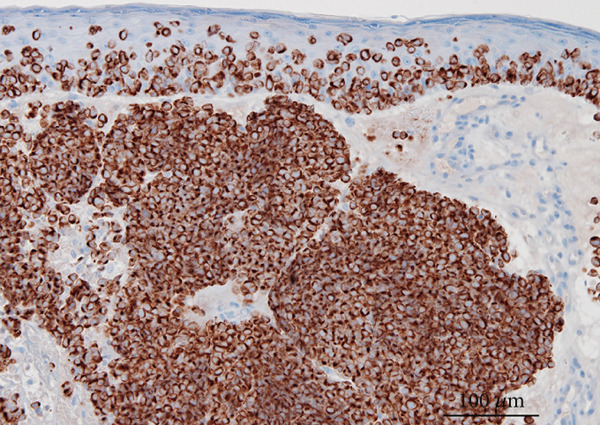
(b)

**Figure:**
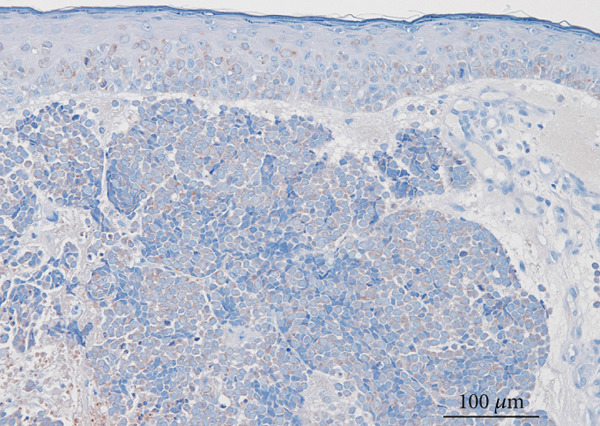
(c)

**Figure:**
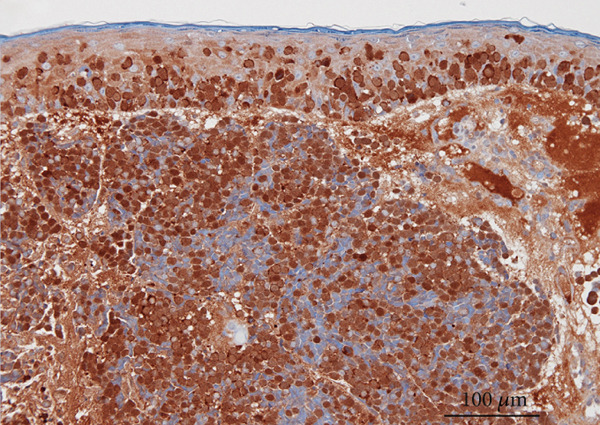
(d)

**Figure 3:**
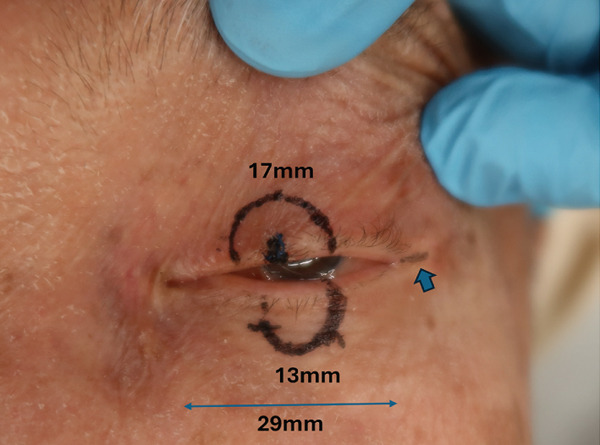
The safety margin was 8 mm, and the eyelid defect was 17 mm. The horizontal diameter of the palpebral fissure width was 29 mm, which made simple suturing difficult, and a 13 mm wide switch flap was created on the lower eyelid. The lateral canthotomy was performed as shown by the blue arrow.

**Figure 4:**
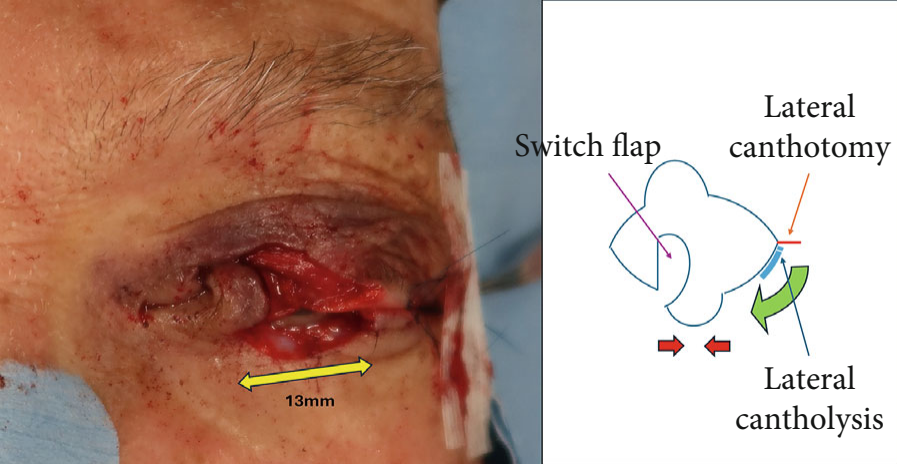
Intraoperative findings. A switch flap was harvested from the lower eyelid and sutured to the upper eyelid. The defect width of the switch flap harvesting site was 13 mm, which made simple suturing difficult, and lateral canthotomy and lateral cantholysis were performed.

No metastases were detected in a positron emission tomography–computed tomography scan, and 13 days after the initial consultation, the patient underwent extensive tumor resection and reconstruction under general anesthesia. The safety margin for the tumor resection was set at 8 mm, and the tumor was resected in all layers of the eyelid. Because the defect in the upper eyelid after tumor resection was 17 mm wide and simple suturing of the defect would be difficult, a switch flap was harvested from the lower eyelid. The switch flap was 3/4 the width of the defect and had a transverse diameter of 13 mm, and it was sutured to the conjunctiva, tarsal plate, and skin of the upper eyelid (Figure 3). Because it was difficult to perform simple suturing of the switch flap donor site, lateral canthotomy and lateral cantholysis were performed, and the ear‐side end of the switch flap donor site was advanced to the nasal side to close the flap donor site (Figure 4).

**Figure 5:**
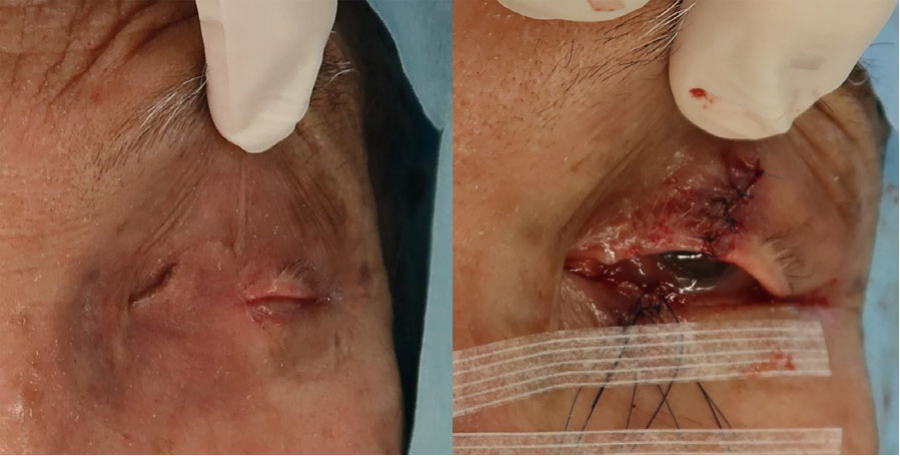
Intraoperative findings during detachment of the flap. The conjunctiva, tarsal plate, and skin within the detached flap were sutured to the corresponding tissues of the upper eyelid.

**Figure 6:**
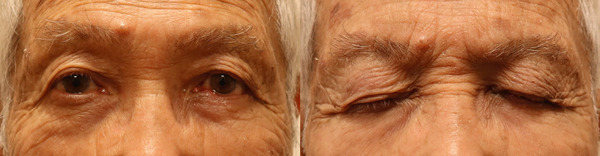
The patient’s condition 6 months after surgery. The eyelid morphology and motility were good, and there were no abnormalities in the corneal findings.

There were no problems with the blood flow to the flap, and the flap was detached under general anesthesia 16 days after the initial surgery (Figure 5). Radiation therapy was not performed because of the risk of corneal ulceration and the patient’s wishes. Six months have passed since the surgery, and there are no problems with the function of the eyelids or the cosmetic appearance. There has been no recurrence or metastasis of the tumor (Figure 6).

## 3. Discussion

Merkel cell carcinoma is a very rare disease that was first reported in 1972 by Toker as trabecular carcinoma of the skin [3]. It occurs commonly in people aged 65 years and over. This carcinoma usually involves exposed areas; the head and neck are the most common sites and account for 45% of cases, 2.5% of which are primary eyelid tumors [1]. Merkel cell carcinoma is also a highly malignant tumor that progresses quickly, and careful and prompt diagnosis and treatment are essential [2]. However, there are various opinions regarding its treatment [4, 5]. The National Comprehensive Cancer Network guidelines recommend a safety margin of 1–2 cm when removing the tumor, but one report notes that local control is possible with a safety margin of 5 mm [4]. However, another report found no correlation between the safety margin and the local recurrence rate [6]. In the case presented here, we struggled to set the safety margin because of the involvement of special areas such as the eyelid. To preserve the functions of the eyelid, such as protecting the eyeball, we set the safety margin at 8 mm, which is the maximum width needed to preserve the lacrimal punctum and inner canthus.

In this case, reconstruction after tumor resection also required some ingenuity because of the special nature of the eyelid. In reconstructing the upper eyelid, simple suturing is possible if the defect is less than one‐quarter of the transverse diameter of the eyelid fissure. However, if the defect is more than one‐quarter of this diameter, suturing becomes difficult, and some type of tissue replacement is necessary [7]. Tissue grafting involving the eyelid requires both maintaining the functions of opening and closing the eyelid to protect the eyeball while retaining the most natural form. Therefore, a switch flap from the lower eyelid (Figure 7a) is often chosen [8].

Figure 7Reconstruction techniques. (a), Reconstruction using a switch flap is shown. The flap harvesting site of the lower eyelid was sutured as shown by the red arrow. (b), If suturing is difficult, the anterior lobe can be reconstructed using a malar flap and the posterior lobe can be reconstructed using a cartilage graft. However, in this case, less invasive surgery was preferred because of the patient’s high risk of bleeding, and lateral canthotomy and lateral cantholysis were performed, as shown in (c). In this procedure, the ear‐side end of the switch flap donor site was advanced to the nasal side to close the flap donor site.

**Figure:**
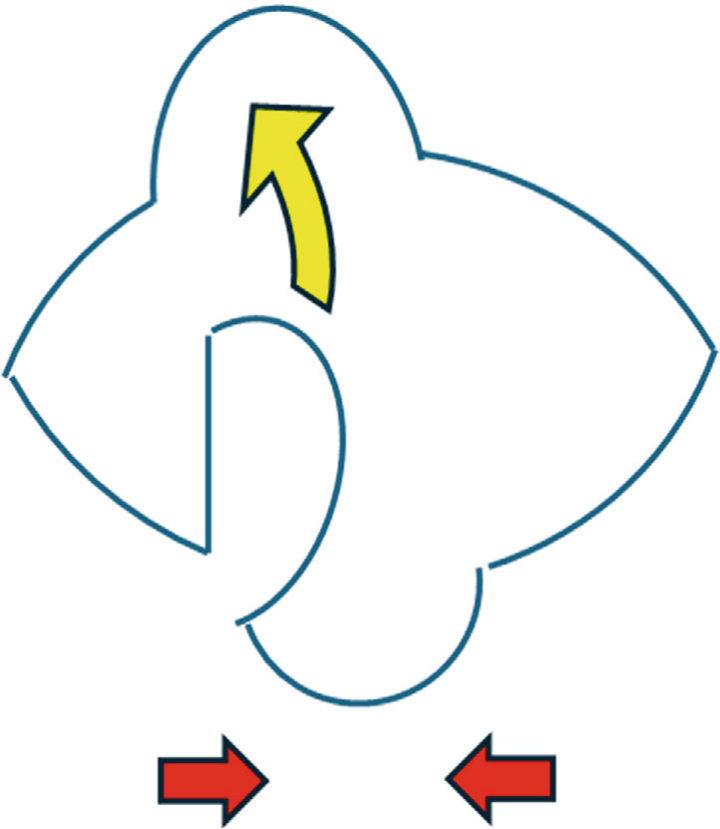
(a)

**Figure:**
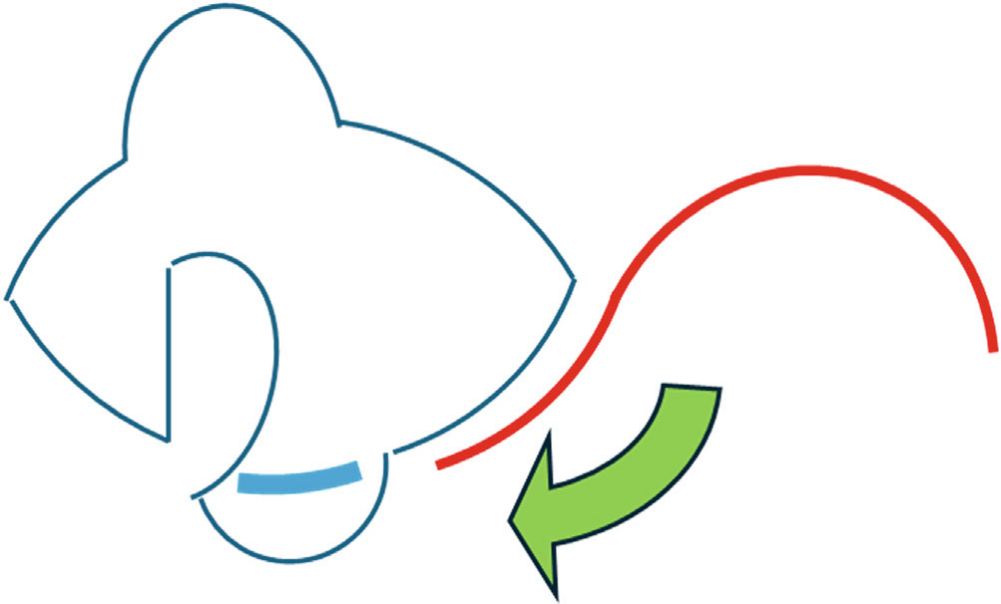
(b)

**Figure:**
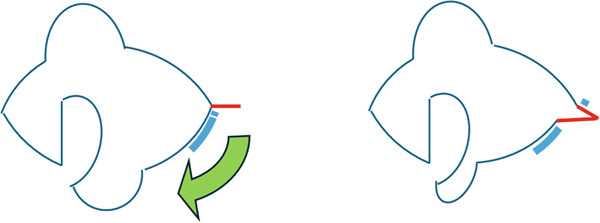
(c)

In the reconstruction of the eyelid using a switch flap, it is difficult to perform simple suturing of the flap harvesting site if the upper eyelid defect exceeds half the width of the palpebral fissure. Therefore, it is common practice to close the switch flap donor site by performing a malar flap for the anterior lobe and a cartilage graft for the posterior lobe (Figure 7b). Baccarani et al. [9] also reported reconstructing a very large 45‐mm upper eyelid defect after MCC excision using a combination of a switch flap and a malar flap. However, donor‐site closure has not been discussed much until recently [8]. In this case, the patient’s palpebral fissure width was 29 mm and the upper eyelid defect width was 17 mm, which made it difficult to close the switch flap donor site with simple sutures. Given the patient’s high risk of bleeding, closure using the abovementioned malar flap and cartilage graft was not performed, and the switch flap harvesting site was closed using a less invasive technique involving the combination of lateral canthotomy and lateral cantholysis (Figure 7c).

Lateral cantholysis and cantholysis have been reported as minimally invasive methods for relaxing the tension of the eyelid [10, 11]. For example, in 1979, Anderson stated that lateral canthotomy and lateral cantholysis can stretch the skin of the lower eyelid [10]. Bruneau reported that when treating orbital floor fractures, cutting the ligament can stretch the lower eyelid and improve vision [11]. This method can also be used for full‐thickness eyelid defects and may be able to advance the eyelid by several millimeters [10], although it is unclear exactly how many millimeters it advances [12].

Milap et al. [13] and Lewis and Perry [14] have reported a method called transconjunctival lateral cantholysis, which is similar to lateral cantholysis and canthopexy, and involves an approach from the conjunctival side without cutting the skin surface. It is believed that this method can advance the eyelid by 4–10 mm and close full‐thickness eyelid defects of 20 mm or more [13]. If the closure is difficult using transconjunctival lateral cantholysis, the next step is reconstruction using a malar flap [14]. Therefore, we believe that the lateral cantholysis and canthopexy method described here is positioned between the direct closure and malar flap methods.

Until now, only two methods for reconstruction using a switch flap have been reported: either direct closure of the flap donor site or closure of the flap donor site using a malar flap [8]. However, we chose to use lateral cantholysis and canthopexy, which fall between these two methods. In the case presented here, the time required for lateral cantholysis and canthopexy was short (5 min), and the amount of bleeding was minimal, and we believe that this method is useful because it is minimally invasive. In terms of cosmetic results, Lewis and Perry [14] reported that creating a wound in the skin around the outer corner of the eye can cause contracture and lead to disfigurement. By contrast, when using our method, we take care to ensure that the incision line and the horizontal line around the outer corner of the eye do not intersect, which prevents contracture, and we believe that the results are satisfactory in terms of cosmetic results.

**Figure 8:**
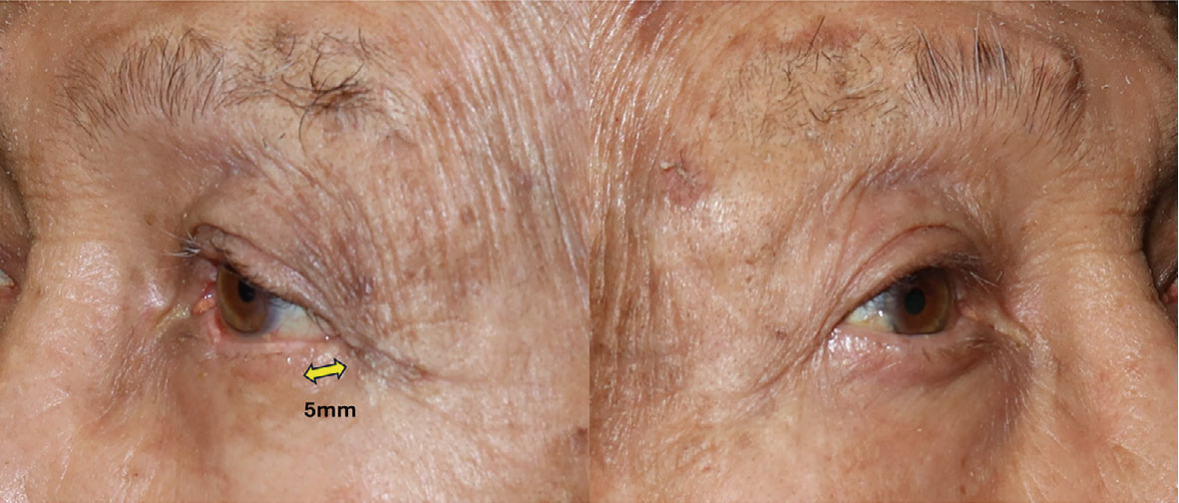
Oblique view taken 6 months after the operation. As indicated by the yellow arrow, a 5 mm area without eyelashes resulted from the lateral canthotomy and lateral cantholysis.

The actual length of advancement using this method and the size of the lower eyelid full‐thickness defect for which this method is suitable should be considered. In this case, the area of the lower eyelid outside the eyelashes after surgery was 5 mm (Figure 8). It appears that a 5 mm advancement was achieved using the lateral canthotomy and lateral cantholysis method, and that the defect width of the flap harvesting area was reduced from 13 to 8 mm, which enabled suturing.

The results presented here suggest that this method may be useful for the reconstruction of full‐thickness upper eyelid defects of up to half the width of the eyelid fissure +5 mm. Given that the average width of an eyelid fissure is 25–30 mm [15], a full‐thickness upper eyelid defect of 13–20 mm is a good indication for this method. Because Merkel cell carcinoma requires extensive excision [2], it is likely to meet the abovementioned criteria if it occurs in the upper eyelid. In addition, this carcinoma occurs most often in elderly people [1], who may have a high risk of bleeding. Therefore, this minimally invasive method may be particularly useful for Merkel cell carcinoma of the upper eyelid.

### 3.1. Statistical Analysis

As this study reports a single patient, no statistical analyses were performed.

## Ethics Statement

The patient has been informed and given his consent to publish the data.

## Consent

Written informed consent for the publication of clinical information and images was obtained from the patient.

## Disclosure

All authors have read and approved the final version of the manuscript. Dr. Ryo Yamochi had full access to all data in this study and takes full responsibility for the integrity and accuracy of the data.

The lead author affirms that this manuscript is an honest, accurate, and transparent account of the study being reported; that no important aspects have been omitted; and that any discrepancies from the study as planned have been explained. As this study reports a single patient, no statistical analyses were performed.

## Conflicts of Interest

The authors declare no conflicts of interest.

## Funding

No funding was received for this manuscript.

## Data Availability

All data supporting the findings of this study are available within the article.

## References

[1] Lemos B. D. , Storer B. E. , Iyer J. G. , Phillips J. L. , Bichakjian C. K. , Fang L. C. , Johnson T. M. , Liegeois-Kwon N. J. , Otley C. C. , Paulson K. G. , Ross M. I. , Yu S. S. , Zeitouni N. C. , Byrd D. R. , Sondak V. K. , Gershenwald J. E. , Sober A. J. , and Nghiem P. , Pathologic Nodal Evaluation Improves Prognostic Accuracy in Merkel Cell Carcinoma: Analysis of 5823 Cases as the Basis of the First Consensus Staging System, Journal of the American Academy of Dermatology. (2010) 63, no. 5, 751–761, 10.1016/j.jaad.2010.02.056, 2-s2.0-77957991808, 20646783. 20646783 PMC2956767

[2] Akhtar S. , Oza K. K. , and Wright J. , Merkel Cell Carcinoma: Report of 10 Cases and Review of the Literature, Journal of the American Academy of Dermatology. (2000) 43, no. 5, 755–767, 10.1067/mjd.2000.106505, 2-s2.0-0033753292, 11050578. 11050578

[3] Toker C. , Trabecular Carcinoma of the Skin, Archives of Dermatology. (1972) 105, no. 1, 107–107, 10.1001/archderm.1972.01620040075020, 2-s2.0-0015250898. 5009611

[4] Peters G. B.3rd, Meyer D. R. , Shields J. A. , Custer P. L. , Rubin P. A. , Wojno T. H. , Bersani T. A. , and Tanenbaum M. , Management and Prognosis of Merkel Cell Carcinoma of the Eyelid, Ophthalmology. (2001) 108, no. 9, 1575–1579, 10.1016/S0161-6420(01)00701-1, 2-s2.0-0034875950, 11535453. 11535453

[5] Harrington C. and Kwan W. , Outcomes of Merkel Cell Carcinoma Treated With Radiotherapy Without Radical Surgical Excision, Annals of Surgical Oncology. (2014) 21, no. 11, 3401–3405, 10.1245/s10434-014-3757-8, 2-s2.0-84922582379, 25001091. 25001091

[6] Soult M. C. , Feliberti E. C. , Silverberg M. L. , and Perry R. R. , Merkel Cell Carcinoma: High Recurrence Rate Despite Aggressive Treatment, Journal of Surgical Research. (2012) 177, no. 1, 75–80, 10.1016/j.jss.2012.03.067, 2-s2.0-84865065177, 22537840. 22537840

[7] Spinelli H. M. and Jelks G. W. , Periocular Reconstruction: A Systematic Approach, Plastic and Reconstructive Surgery. (1993) 91, no. 6, 1017–1024, 10.1097/00006534-199305000-00007, 2-s2.0-0027312080. 8479966

[8] Mustardé J. C. , Major Reconstruction of the Eyelids: Functional and Aesthetic Considerations, Clinics in Plastic Surgery. (1981) 8, no. 2, 227–236, 10.1016/S0094-1298(20)30449-1, 7273625. 7273625

[9] Baccarani A. , Pompei B. , Pedone A. , and Brombin A. , Merkel Cell Carcinoma of the Upper Eyelid: Presentation and Management, International Journal of Oral and Maxillofacial Surgery. (2013) 42, no. 6, 711–715, 10.1016/j.ijom.2012.10.031, 2-s2.0-84877025588, 23219709. 23219709

[10] Anderson R. L. and Gordy D. D. , The Tarsal Strip Procedure, Archives of Ophthalmology. (1979) 97, no. 11, 2192–2196, 10.1001/archopht.1979.01020020510021, 2-s2.0-0018728677. 508189

[11] Bruneau S. and Scolozzi P. , Preseptal Transconjunctival Approach to the Orbital Floor Fractures. Surgical Technique, Revue de Stomatologie, de Chirurgie Maxillo-Faciale et de Chirurgie Orale. (2015) 116, no. 6, 362–367, 10.1016/j.revsto.2015.10.004, 2-s2.0-84951799148, 26586596. 26586596

[12] Tomassini G. M. , Ricci A. L. , Covarelli P. , Cencetti F. , Ansidei V. , Rulli A. , and Simonetti S. , Surgical Solutions for the Reconstruction of the Lower Eyelid: Canthotomy and Lateral Cantholisis for Full-Thickness Reconstruction of the Lower Eyelid, In Vivo. (2013) 27, no. 1, 141–145, 23239863. 23239863

[13] Milap P. M. , Lewis C. D. , and Perry J. D. , Internal Cantholysis for Full Thickness Eyelid Defects, Saudi Journal of Ophthalmology. (2011) 25, no. 1, 31–36, 10.1016/j.sjopt.2010.10.007, 2-s2.0-78650767980. 23960900 PMC3729649

[14] Lewis C. D. and Perry J. D. , Transconjunctival Lateral Cantholysis for Closure of Full-Thickness Eyelid Defects, Ophthalmic Plastic & Reconstructive Surgery. (2009) 25, no. 6, 469–471, 10.1097/IOP.0b013e3181b80ff8, 2-s2.0-73949147432, 19935251. 19935251

[15] Bergin D. J. , McCord C. D. , Tanenbaum M. , and Nunery W. , Anatomy of the Eyelids, Lacrimal System, and Orbit, Oculoplastic Surgery, 1995, 3rd edition, Raven Press, 51–84.

